# methylMapR—an R package to generate the functional prokaryotic methylome

**DOI:** 10.1128/mra.01240-24

**Published:** 2025-05-20

**Authors:** Christopher Morrissey, Arun Sethuraman

**Affiliations:** 1 Department of Biology, San Diego State University 115105, San Diego, California, USA; 2 Illumina Inc. 10908, San Diego, California, USA; Loyola University Chicago, Chicago, Illinois, USA

**Keywords:** software, methylation, prokaryotes

## Abstract

Here, we present an R package called methylMapR to capture the functional methylome from long read sequencing of prokaryotic genomes using the PacBio sequencing platform. We then describe its utility by comparative analyses of the functional methylomes in three bacterial species*—Escherichia coli*, *Klebsiella pneumoniae*, and *Pseudomonas aeruginosa*.

## ANNOUNCEMENT

DNA methylation has been studied extensively in transcription regulation and gene expression across prokaryotes (1–4). A shared aspect of prokaryotic DNA methylation is that DNA methyltransferase (DNMT) requires the methyl donor molecule, S-adenosyl methionine, and covalently transfers the heritable epigenetic methyl tag to a base within its target motif (5). During binary fission, each daughter chromosome is hemi-methylated with the daughter strand retaining methylation patterns from its parent chromosome. The nascent strand remains unmethylated, needing to be remethylated by DNMT (6). Active gene processes like transcription can block or modulate the methylation reaction during re-methylation events (7). Conversely, methylation has been shown to both promote and repress transcription. Some technologies like PacBio Sequencing, henceforth PBS, and its associated software, *kineticsTools*, can monitor polymerase kinetics by tracking the time of incorporation of each new base on the nascent strand and estimate the interpulse duration ratio (IPD), the likelihood that a methyl functional group is present at that base (8, 9). A high IPD ratio is indicative of a slower incorporation rate by DNA polymerase, which can be caused by the presence of a methyl group (10, 11). Here, we present methylMapR, a tool that leverages the IPD ratio data from PBS and combines it with other functional genomic data to describe the *functional prokaryotic methylome*.

**Fig 1:**
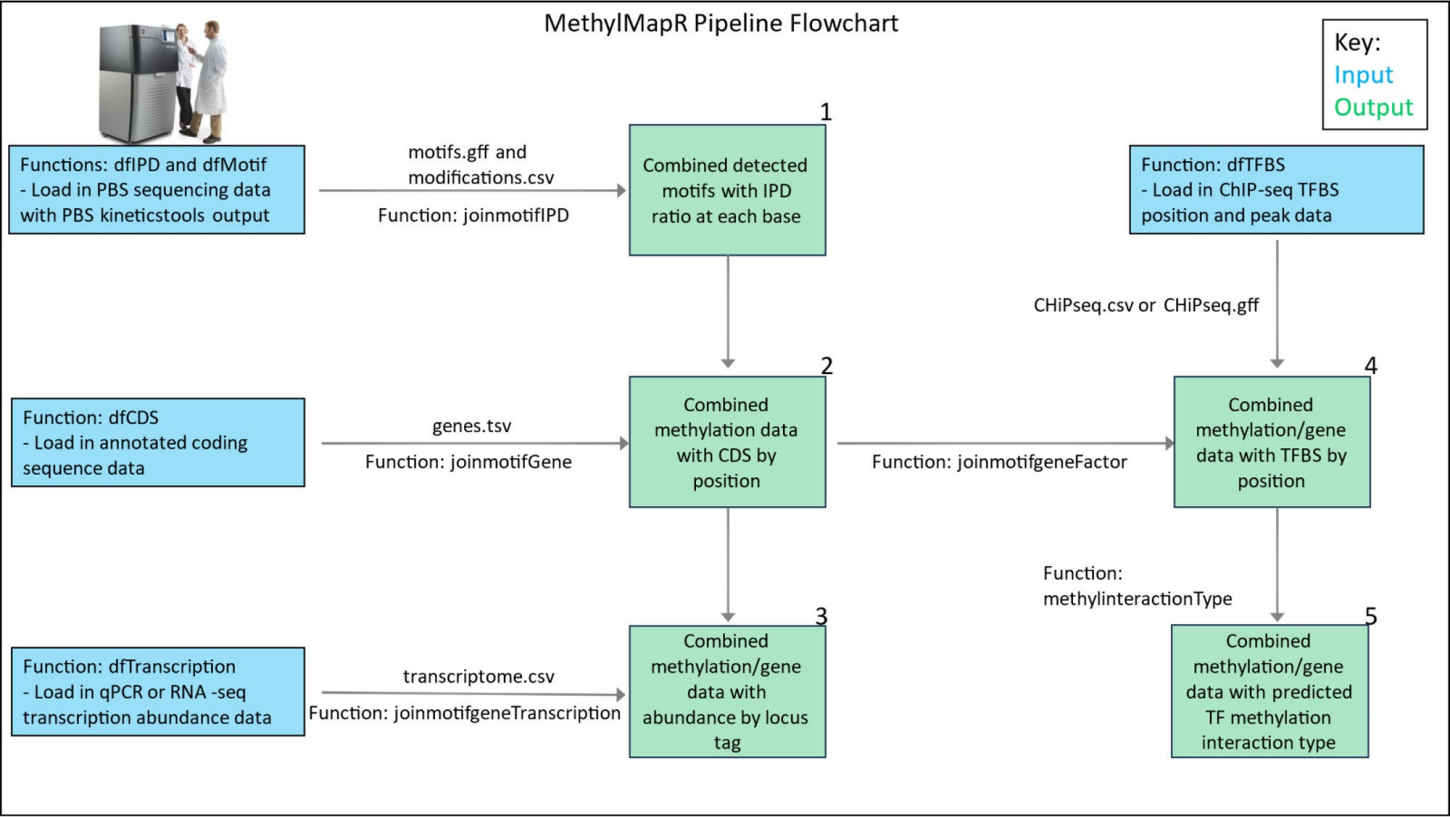
A flowchart showing inputs and outputs generated by functions in the methylMapR R package.

methylMapR implements several mapping functions that calculate new metrics and/or add columns to the output data frames, including assigning transcription factor methylation interaction type, finding promoter-associated motif sites, and calculating the number of TFBSs near each motif (Fig. 1). We utilized and compared functional methylomes from publicly available NCBI repositories for three well-studied prokaryotes, *Escherichia coli*, specifically the laboratory-born K12 strain, *Klebsiella pneumoniae*, and *Pseudomonas aeruginosa*. We acknowledge that none of these data comes from a single source or experiment(s). Our analyses here were therefore designed to help compare the power of methylMapR across multiple genera.

A comparison of the output from methylMapR and summary metrics of the three functional methylomes regarding transcription is presented in Table 1. The global IPD ratio observed across methylomes suggested that *E. coli* had the highest average IPD, for all known methylation types (m4C: 1.0657, m5C: 1.1643, and m6A: 1.1945). Global IPD ratios in both *K. pneumoniae* and *P. aeruginosa* were lower, exhibiting 1.1001 and 1.0018 IPD ratio at target adenines with a m6A motif, respectively. Comparing the types and proportions of motifs that were methylated, we observe one main difference between the isolates*—E. coli* has an extra methyl transferase/motif type (m5C) that the other two prokaryotes do not. Additionally, we uncovered differences in proportions of detected methylation motifs across all species. *K pneumoniae* shows over 60% of its methylation motifs are from the m6A tag. The m4C tag, though present in all species, accounts for less than 5% of all motifs in all three methylomes. All methylomes also show interactions with transcription factor binding sites, and the repressive methylation type was the most common interaction predicted. None of the methylomes had a methylation promotion rate above 20% at all methylated motif sites.

**TABLE 1:** Methylation/transcription summary metrics estimated from the methylMapR pipeline in three prokaryotic species*—E. coli*, *K. pneumoniae*, and *P. aeruginosa*, by methylation type^*a*^

Prokaryote	IPD ratio at methylation target bases and all other bases	Number and proportion of methylation types	Methylation motif/TFBS crowdedness (10 bp window, both strands) Avg and Max	Number and proportion of promoter region-associated motifs	TFBS/methyl interaction types – ratio of promoting to repressive
*E. coli*	m4C: 1.0657 m5C: 1.1643 m6A: 1.1945 modified base: 1.0656 non-target base: 1.0679	m4C: 3215, 0.0236 m5C: 25187, 0.1848 m6A: 40168, 0.2947 modified base: 67721, 0.4969	m4C: 1.4325, 4 m5C: 1.4406, 6 m6A: 1.4075, 4 modified base: 1.4324, 6	m4C: 27, 0.0260 m5C: 212, 0.2040 m6A: 325, 0.3128 modified base: 475, 0.4572	m4C: 62:445, 0.1393 m5C: 784:6381, 0.1229 m6A: 1592:11919, 0.1336 modified base: 941:7380, 0.1275
*K. pneumoniae*	m4C: 1.0137 m6A: 1.1001 modified base: 1.0398 non-target base: 0.9735	m4C: 3464, 0.0297 m6A: 77724, 0.6666 modified base: 35415, 0.3037	m4C: 2.0370, 6 m6A: 2.030791, 6 modified base: 2.059907, 6	m4C: 50, 0.0302 m6A: 1041, 0.6286 modified base: 565, 0.3412	m4C: 21:93, 0.2258 m6A: 705:4622, 0.1525 modified base: 184:1275, 0.1443
*P. aeruginosa*	m4C: 1.0523 m6A: 1.0018 modified base: 1.0403 non-target base: 1.0143	m4C: 3126, 0.0398 m6A: 21121, 0.2692 modified base: 54216, 0.6910	m4C: 1.0000, 1 m6A: 1.0364, 2 modified base: 1.0159, 2	m4C: 19, 0.0294 m6A: 178, 0.2755 modified base: 449, 0.6950	m4C: 0:0, 0 m6A: 2:3, 0.6667 modified base: 4:5, 0.8000

^*a*^ For TFBS/methyl interaction types – ratio of promoting to repressive” column, only those that are likely modified are included. Definition of “modified base”: any base with detected methylation signal, that has no known target motif. For “methylation motif/TFBS crowdedness (10 bp window, both strands) Avg and Max” column, only those motifs with a TFBS are included in the calculation. For *P. aeruginosa*, the peakHeight metric was on a different scale than for the other two organisms, so a peakHeight parameter of 150 was used, instead of 10. For *P. aeruginosa* TFBS crowdedness data, the ChIP-seq data are one sided, and it includes both strands for the other organisms. peakHeight value is the height of the chipSeq peak from a transcription factor binding assay. These data are merged in the pipeline’s last step to the methylation data from PacBio. Its value (cutoff) can change based on the chipSeq data used, and how that was created. It is the size of the signal at a certain binding position. If peaks are very small, they could not be a true binding event, or binding was not as strong due to mismatches. These values will therefore have to be adjusted on a per run basis, based on the distribution from the assay, and the guidance for that assay. But, users would want to use the higher confidence chipseq spots, rather than all of them.

The methylMapR R package is available at https://github.com/therealtotodile/methylMapR/ and was tested on R 4.4.2 GUI 1.81 Big Sur ARM build (8462). To install methylMapR, source code, example data sets and code outputs, users can download the .gz to run on MacOSX, Windows, or Linux. From RStudio, users can go to Tools->Install packages, and under Install From , select the methylMapR Package Archive File (.tar.gz), then click install. R code to replicate analyses described in this manuscript is available via FigShare DOI: 10.6084/m9.figshare.2858581710.6084/m9.figshare.28585817.

## Data Availability

The data is publicly available on: https://www.ncbi.nlm.nih.gov/genbank/basemodificationfiles/ https://www.ncbi.nlm.nih.gov/datasets/gene/taxon/511145/, GCF_000240185.1/, GCF_000006765.1/, GEO: GSE122295, GSE159777, GSE35926, GSE128430, GSE65356. Other: https://journals.asm.org/doi/10.1128/msystems.00202-19, https://www.pnas.org/doi/full/10.1073/pnas.1717525115#supplementary-materials).
